# Alveolar Ridge Regeneration Using Flexible Allogeneic Laminar Bone for Implant Site Development: A Case Series

**DOI:** 10.3390/dj14090600

**Published:** 2026-09-16

**Authors:** Edgar Salas, Gregori M. Kurtzman

**Affiliations:** 1 Private Practice, Cúcuta 20906, Colombia; edgarsalasodcx@hotmail.com; 2 Private Practice, Silver Spring, MD 20906, USA

**Keywords:** guided bone regeneration, alveolar ridge augmentation, flexible allogeneic laminar bone, allograft, implant site development, horizontal ridge defect, ridge preservation, dental implants

## Abstract

**Background:** Horizontal alveolar ridge deficiencies resulting from tooth loss, implant failure, trauma, or advanced ridge atrophy frequently require augmentation before prosthetically driven implant placement can be achieved. Flexible allogeneic cortical bone laminae have been introduced as biologic containment devices that also eliminate the need for titanium mesh or non-resorbable barrier membranes. **Methods:** Three patients presenting with horizontal ridge deficiencies were treated using guided bone regeneration (GBR) with flexible allogeneic laminar bone combined with particulate mineralized and demineralized cortical allografts. The laminar bone was adapted beneath full-thickness flaps to ensure semi-rigid containment of the graft material and maintenance of the regenerative space. Clinical and radiographic healing were evaluated using periapical radiographs and cone beam computed tomography (CBCT). Implants were placed following healing, and patients were followed up with for up to three years. **Results:** All treated sites demonstrated successful horizontal ridge augmentation with preservation of ridge contours and sufficient bone volume for implant placement. Primary implant stability of at least 35 Ncm was achieved in the documented cases. CBCT evaluation confirmed bone formation along the buccal aspect of the implants and maintenance of ridge dimensions. No graft collapse, membrane exposure, infection, or donor-site morbidity was observed. Long-term follow-ups demonstrated stable peri-implant hard- and soft-tissue conditions. **Conclusions**: Flexible allogeneic laminar bone used in combination with a particulate allograft appears to be a predictable and minimally invasive approach for horizontal ridge augmentation and implant site development. The technique offers effective space maintenance, favorable ridge contour preservation, and stable regenerative outcomes while reducing surgical morbidity associated with traditional augmentation procedures. This report demonstrates clinical feasibility and that larger prospective controlled studies with quantitative outcome assessments are needed to confirm these preliminary observations.

## 1. Introduction

Alveolar ridge resorption following tooth extraction remains a significant challenge in implant dentistry. Physiological remodeling of extraction sites results in a progressive decrease in ridge width and height, particularly along the buccal aspect of the alveolar process. These dimensional changes may compromise ideal implant positioning, adversely affect esthetic outcomes, and increase the need for ridge augmentation procedures prior to implant placement [1,2,3,4].

Numerous clinical and preclinical investigations have demonstrated that the majority of post-extraction remodeling occurs within the buccal plate. Factors such as initial buccal bone thickness, soft-tissue phenotype, and ridge morphology significantly influence the magnitude of these dimensional changes [5,6,7,8,9,10]. Consequently, preservation or reconstruction of the alveolar ridge has become an essential component of contemporary implant treatment planning.

Guided bone regeneration (GBR) is a well-established technique for the treatment of localized alveolar defects and implant site development. The biological principle of GBR involves preventing rapidly proliferating soft-tissue cells from entering the regenerative site while maintaining a protected environment that promotes angiogenesis, migration of osteogenic cells, and new-bone formation. Over the past three decades, GBR procedures have demonstrated predictable clinical outcomes in both ridge preservation and ridge augmentation applications [11].

Traditional GBR procedures frequently involve the use of non-resorbable barrier membranes, titanium-reinforced membranes, or titanium mesh to maintain regenerative space and stabilize graft materials. Although these approaches have demonstrated favorable outcomes, they are associated with several disadvantages, including increased surgical complexity, a risk of membrane exposure, patient discomfort, and the frequent need for a second surgical procedure for device removal [11]. In contrast, resorbable collagen membranes eliminate the need for retrieval but may provide limited structural support in relation to larger horizontal defects.

**Figure 1:**
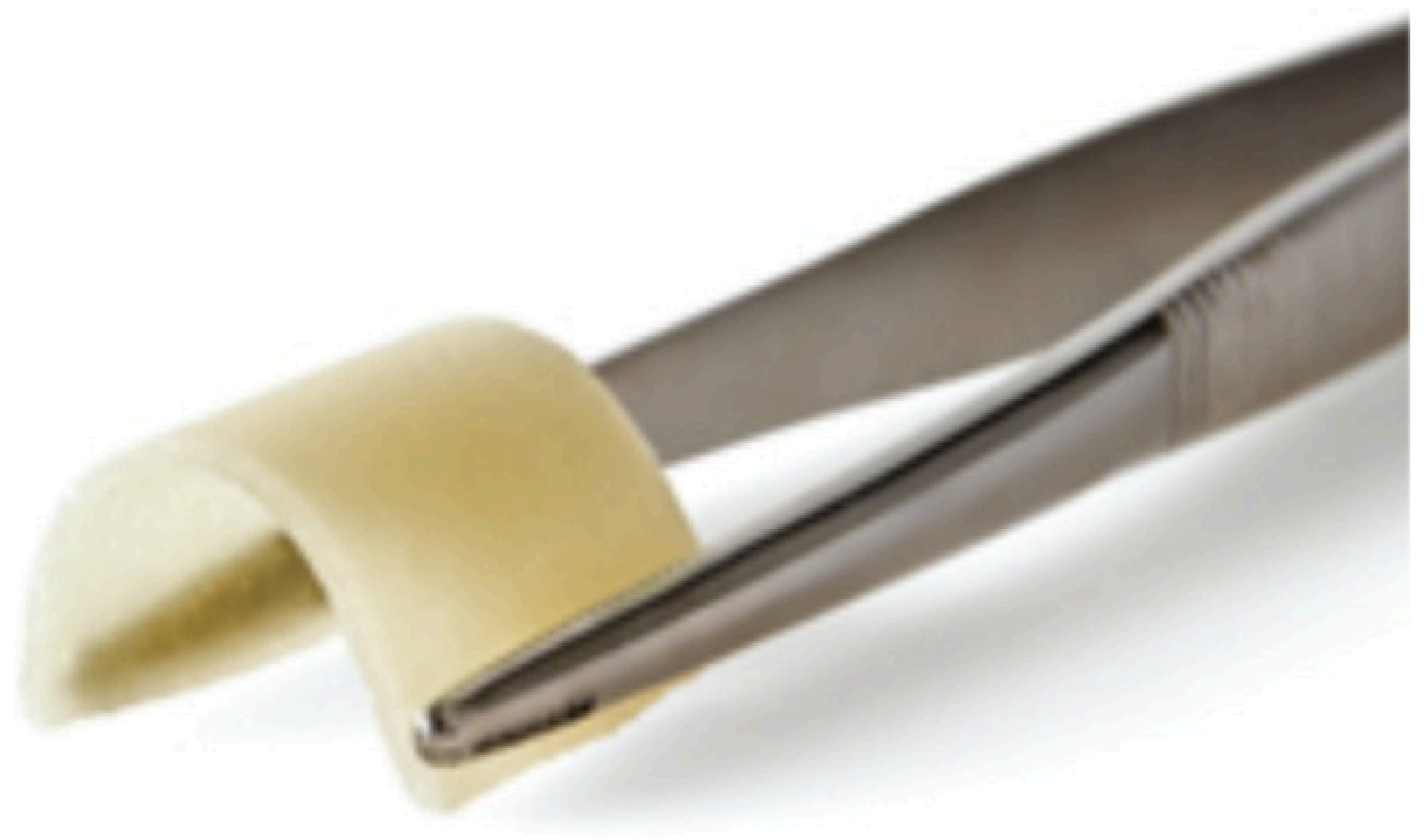
Flexible allogeneic laminar bone supplied in sterile saline and ready for clinical adaptation.

To overcome these limitations, alternative biological containment devices have been developed to combine the advantages of space maintenance with gradual integration into the recipient site. Flexible allogeneic laminar bone (Maxxeus Dental, Kettering, OH, USA) is manufactured from processed cortical bone and retains sufficient flexibility to permit adaptation to complex osseous contours while maintaining structural integrity (Figure 1). Unlike conventional collagen membranes, flexible laminar bone provides semi-rigid support to particulate graft materials, helping to preserve regenerative space and resist soft-tissue collapse during healing.

When used in combination with a particulate allograft, flexible allogeneic laminar bone may provide a biologically favorable environment for ridge reconstruction while avoiding the morbidity associated with autogenous block grafting and the additional surgical procedures often required for non-resorbable barrier systems. The purpose of this case series is to evaluate the clinical application of flexible allogeneic laminar bone in combination with particulate allografts for horizontal ridge augmentation and implant site development in patients presenting with localized alveolar ridge deficiencies.

### Patient Selection

Three consecutive patients requiring horizontal alveolar ridge augmentation prior to implant placement were treated using flexible allogeneic laminar bone in combination with a particulate allograft. The inclusion criteria were as follows: having localized horizontal ridge deficiencies that prevent prosthetically driven implant placement without augmentation, having enough soft-tissue coverage to permit primary closure, and being willing to return for a scheduled follow-up. All patients underwent comprehensive clinical and CBCT evaluation before treatment planning.

Patients who had uncontrolled systemic diseases known to impair wound healing, untreated active periodontal disease, uncontrolled diabetes, or immunosuppressive conditions; had undergone previous head-and-neck radiation therapy; or were taking medications associated with impaired bone healing were not considered candidates for this technique. Users who heavily used tobacco were excluded, and any history of smoking was documented as part of a patient’s medical history. Prior to ridge augmentation, all patients demonstrated satisfactory plaque control and periodontal stability. Each patient provided written informed consent before treatment.

For each case presented, the patient signed a consent form prior to initiation of treatment per Columbia’s ethics requirements for the treatment and publication of anonymized clinical data and clinical/radiographic images. Consent was obtained from all patients according to the applicable national regulations. Formal Institutional Review Board (IRB)/Ethics Committee approval was not required for this descriptive case series. In addition, written informed consent for the treatment and publication of anonymized clinical data and clinical/radiographic images was obtained from all patients.

## 2. Case 1

**Figure 2:**
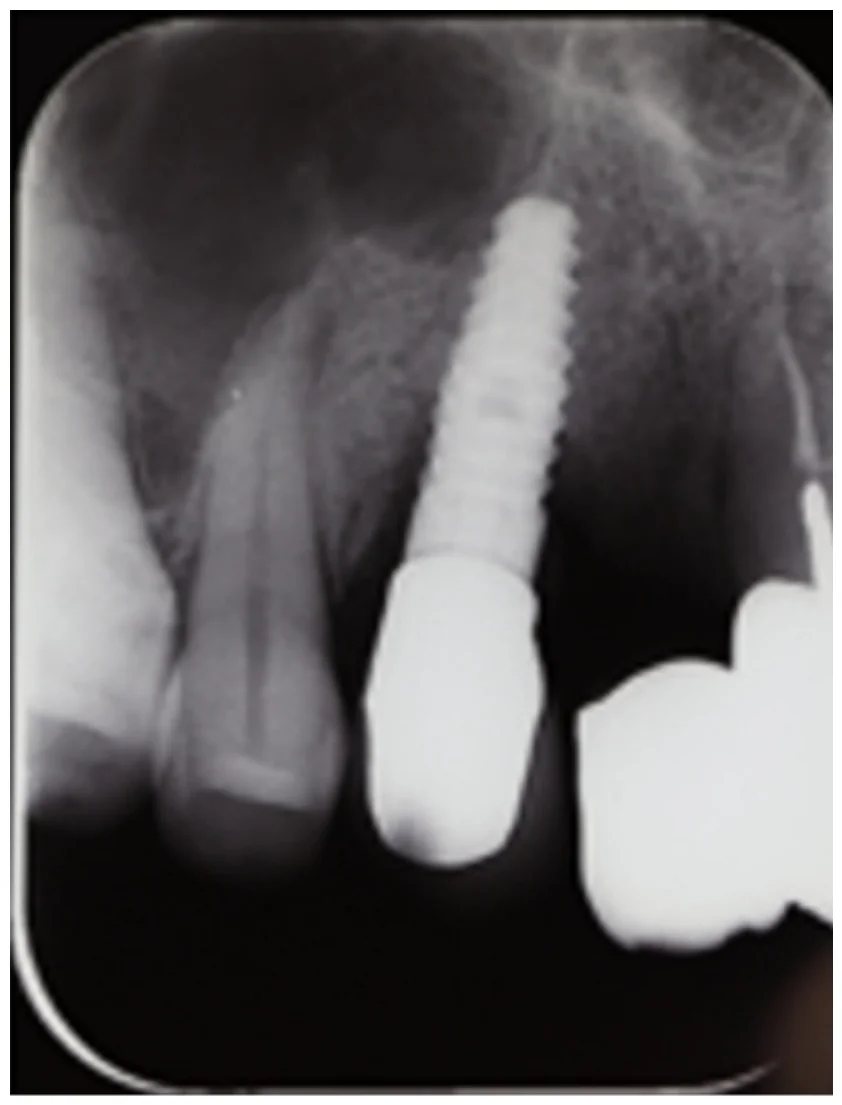
Preoperative periapical radiograph demonstrating extensive peri-implant bone loss associated with the failing implant in the maxillary left-canine region.

**Figure 3:**
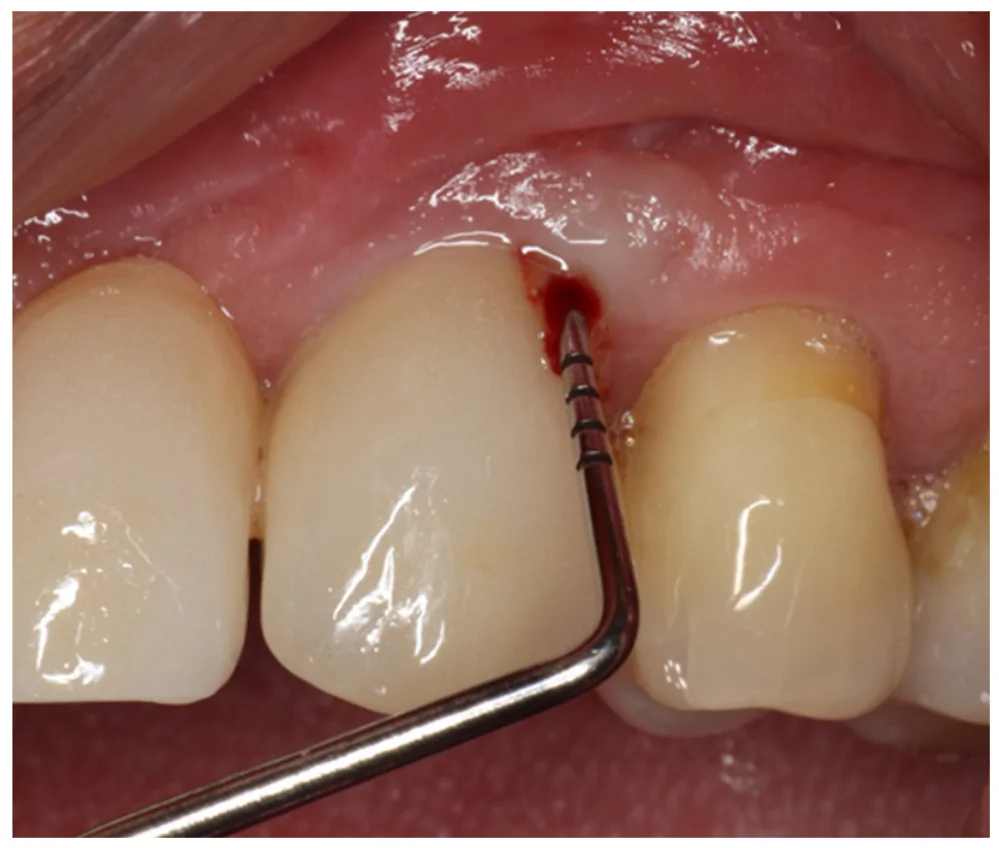
Clinical examination revealing a 15 mm peri-implant probing depth with bleeding on probing, suppuration, and implant mobility.

A 56-year-old male with no significant medical history presented with pain, suppuration, and mobility associated with a dental implant in the maxillary left-canine region. Radiographic evaluation demonstrated a peri-implant radiolucency suggestive of advanced peri-implant bone loss (Figure 2). Clinical examination revealed a 15 mm peri-implant probing depth with bleeding on probing, along with suppuration and implant mobility (Figure 3). Based on the clinical and radiographic findings, the implant was diagnosed as failing and scheduled for removal followed by site reconstruction to facilitate future implant replacement.

**Figure 4:**
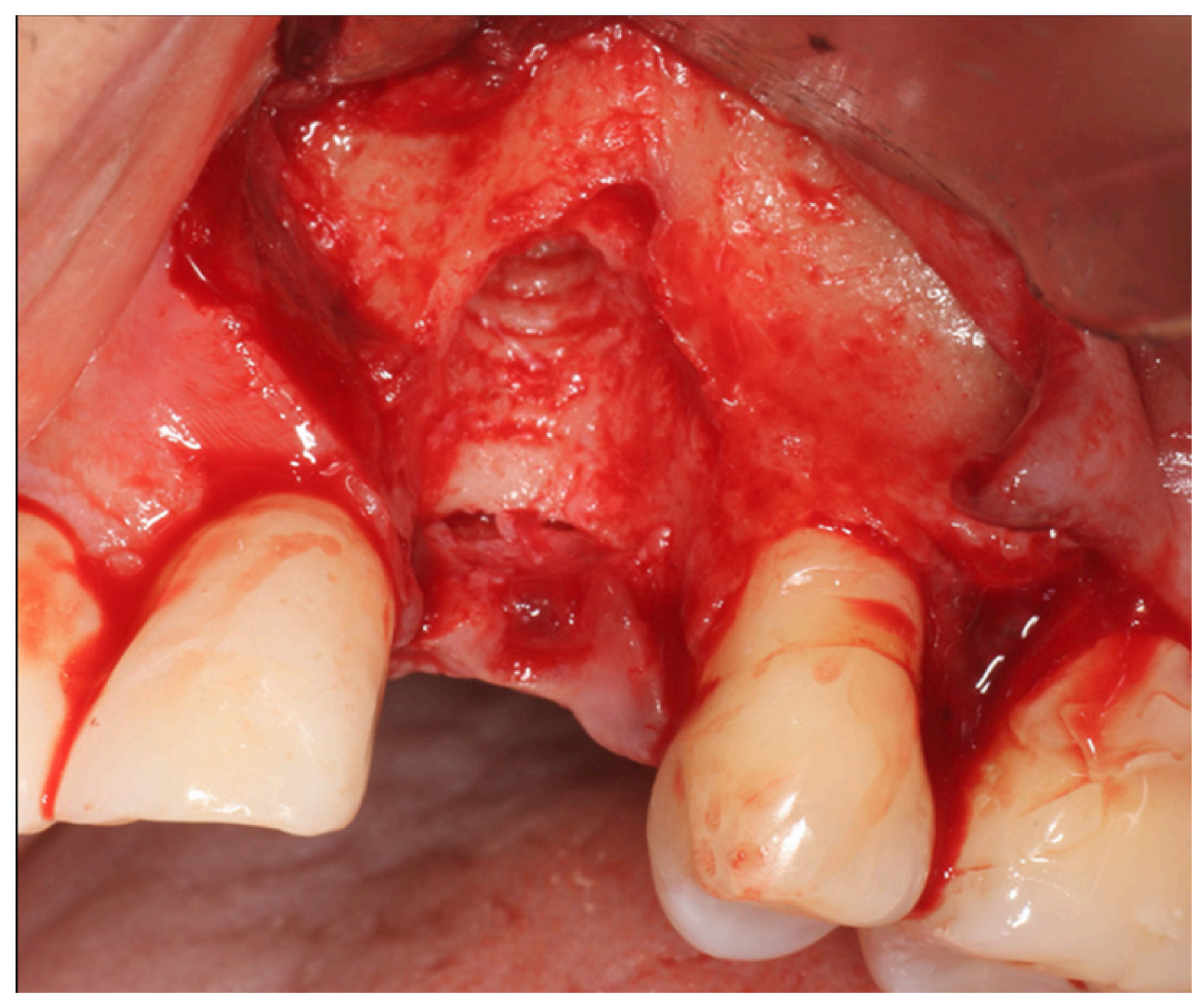
Surgical exposure following flap reflection and implant removal demonstrating significant loss of the buccal plate.

Following administration of local anesthesia, a full-thickness mucoperiosteal flap was elevated using a crestal incision with a mesial releasing incision and palatal sulcular extension. The implant was explanted, and the defect was thoroughly debrided and decontaminated using curettes, saline irrigation, and diode laser therapy [12] (Figure 4).

**Figure 5:**
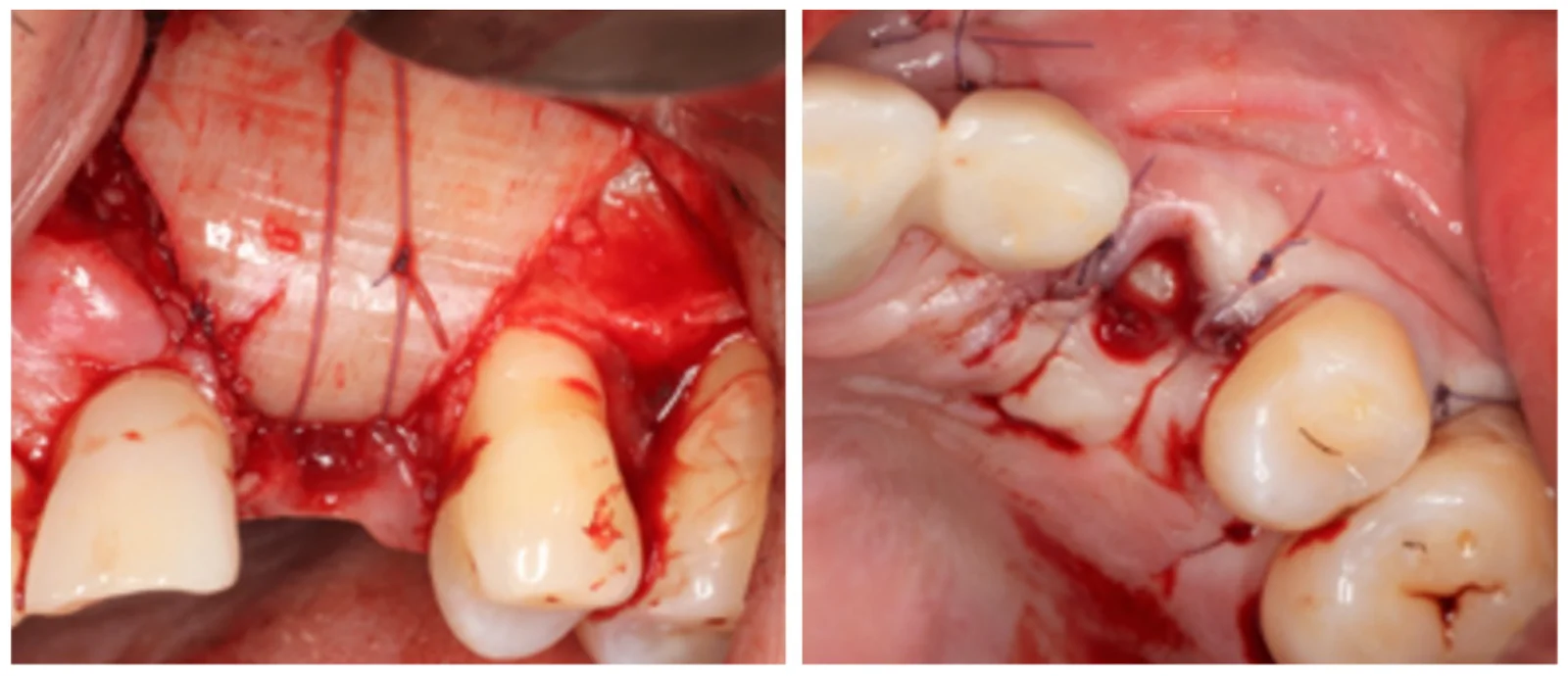
Placement of particulate allograft within the defect followed by adaptation and stabilization of flexible laminar bone using resorbable sutures (**left**). Flap repositioning and closure following graft placement (**right**).

Flexible allogeneic laminar bone, supplied in sterile saline, was trimmed to the dimensions of the defect using a previously fabricated template. A particulate graft consisting of a cortical–cancellous allograft (50:50) combined with a mineralized/demineralized cortical allograft (70:30) was hydrated with sterile saline and packed into the defect. The laminar bone was then adapted over the grafted site and stabilized to the periosteum using 5-0 resorbable sutures, creating a biologic containment structure that maintained graft stability and regenerative space (Figure 5).

**Figure 6:**
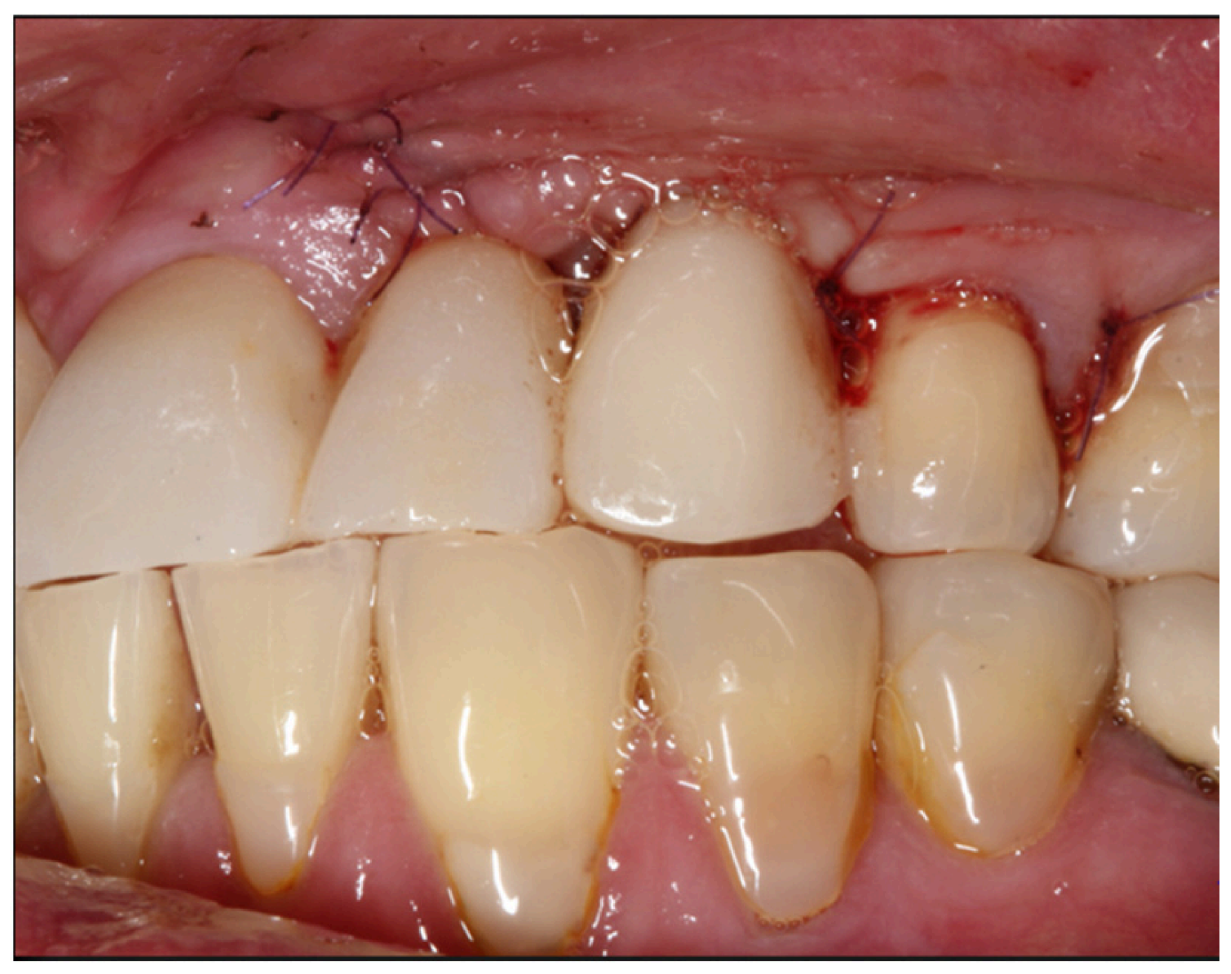
Bonded provisional restoration placed on adjacent teeth to support soft-tissue contours and preserve gingival architecture during healing.

The flap was repositioned without extensive periosteal release, preserving vestibular depth while achieving tension-free closure. A bonded provisional restoration was applied to the adjacent teeth to support the soft tissues and maintain gingival architecture during healing (Figure 6).

**Figure 7:**
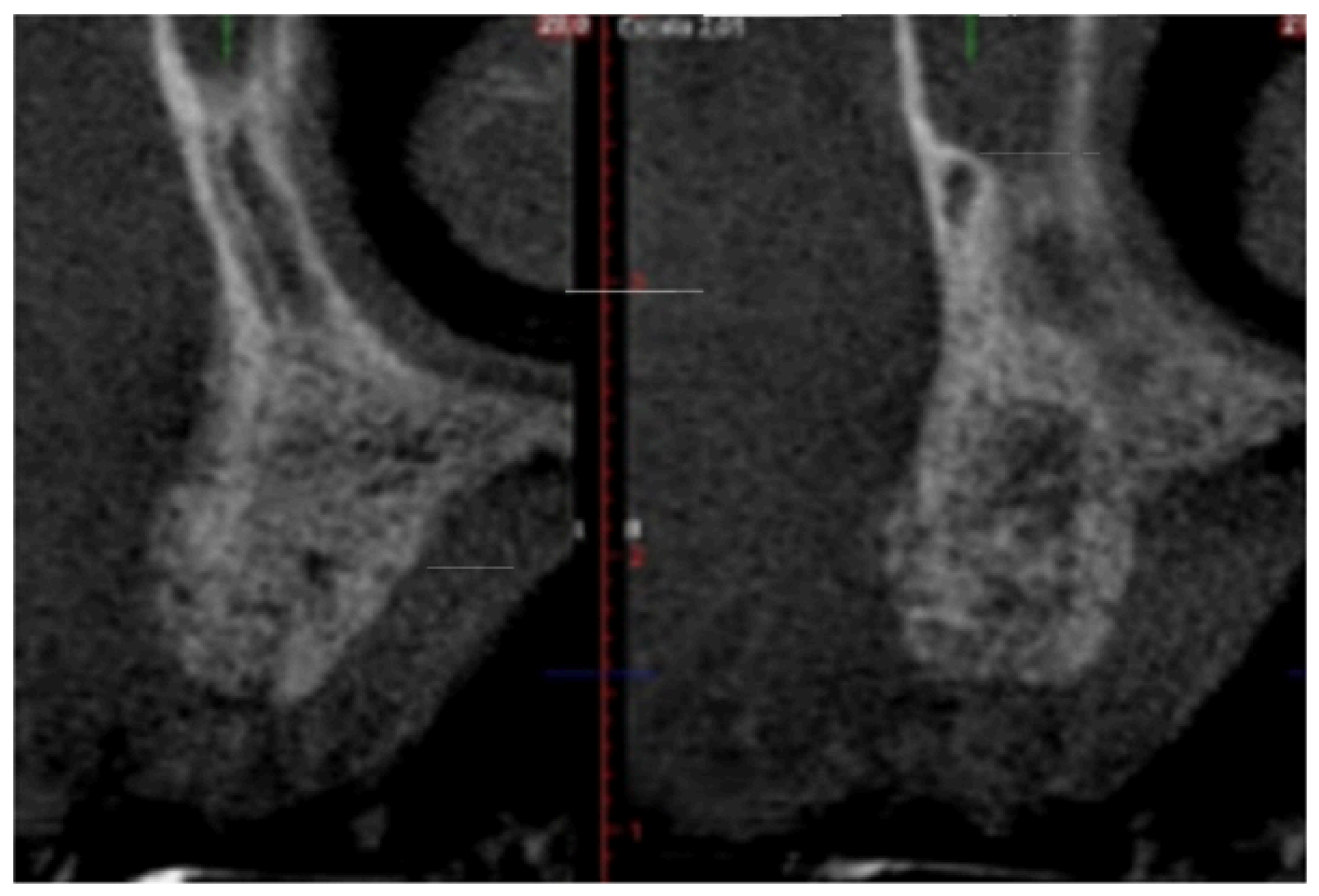
Four-month postoperative CBCT cross-sectional image demonstrating regeneration of ridge width and maturation of grafted bone.

**Figure 8:**
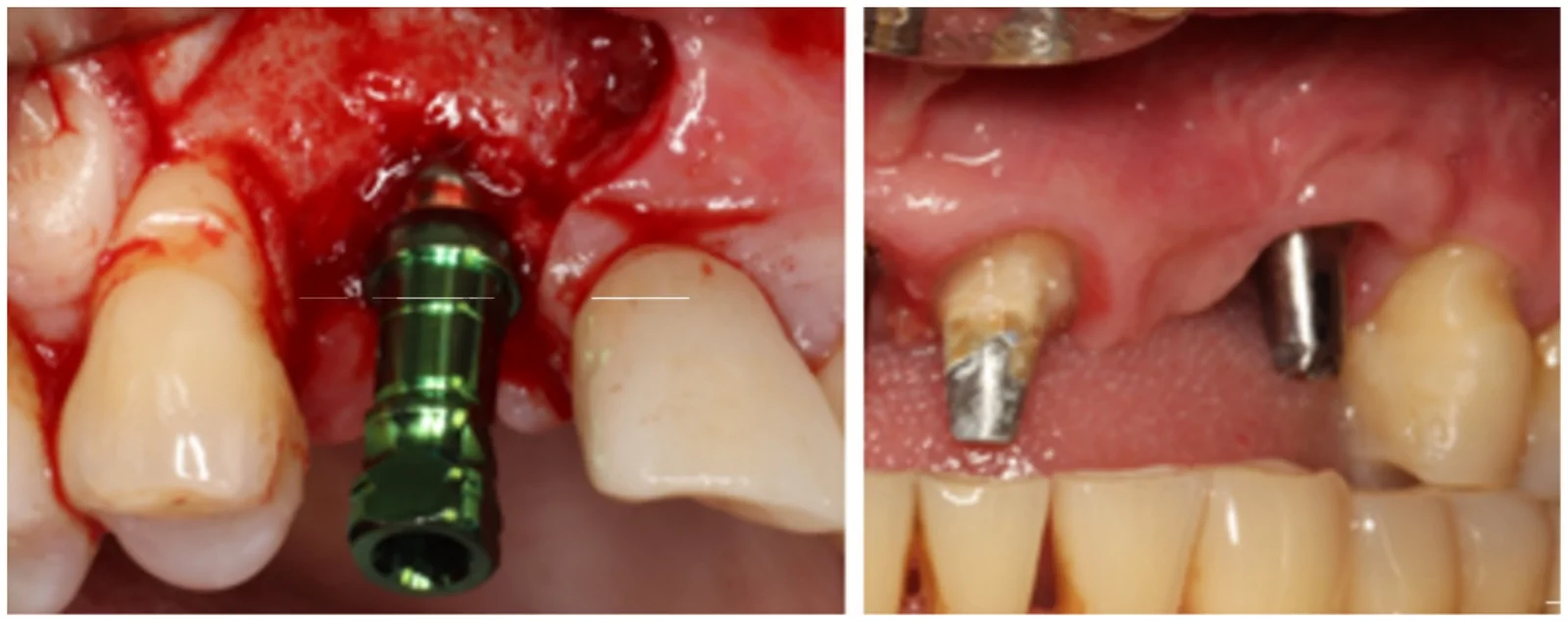
Implant placement following osteotomy preparation with achievement of primary stability (**left**). Implant exposure and initiation of restorative treatment after healing (**right**).

**Figure 9:**
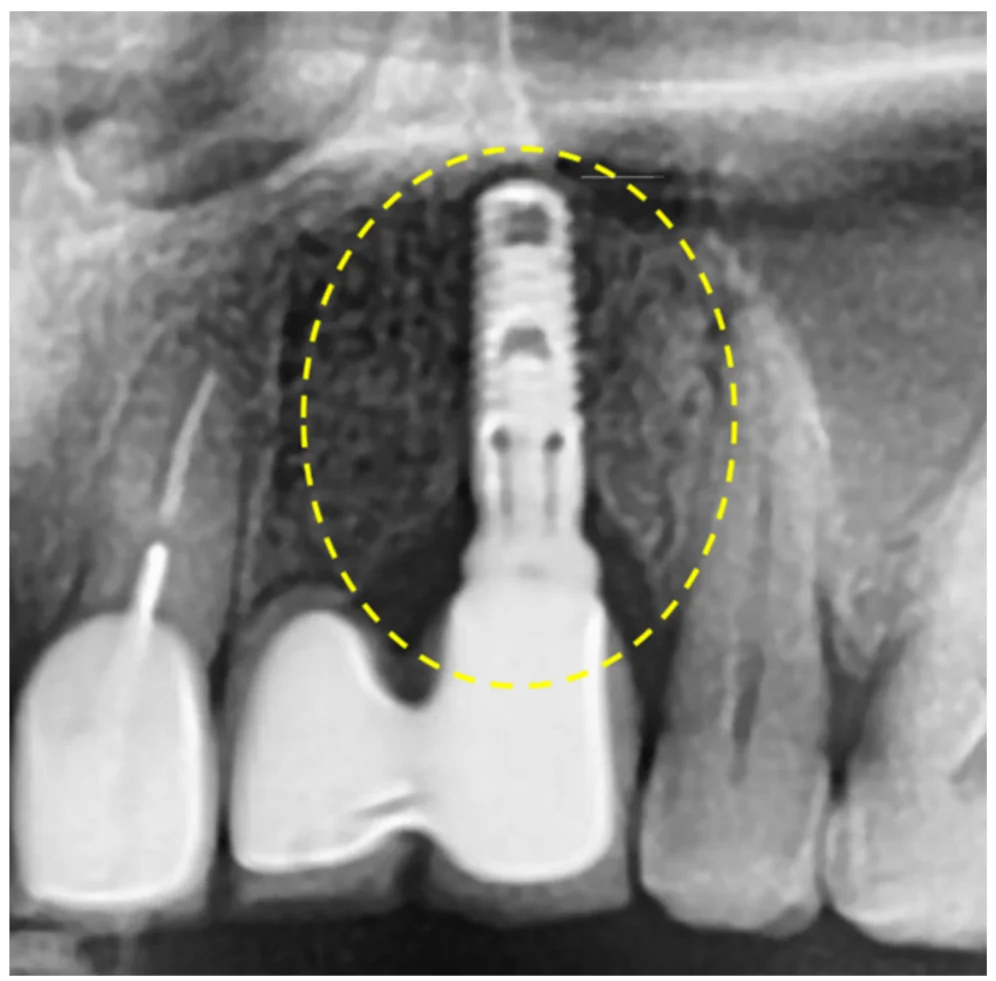
One-year postoperative radiograph demonstrating maintenance of peri-implant bone levels and ridge dimensions.

**Figure 10:**
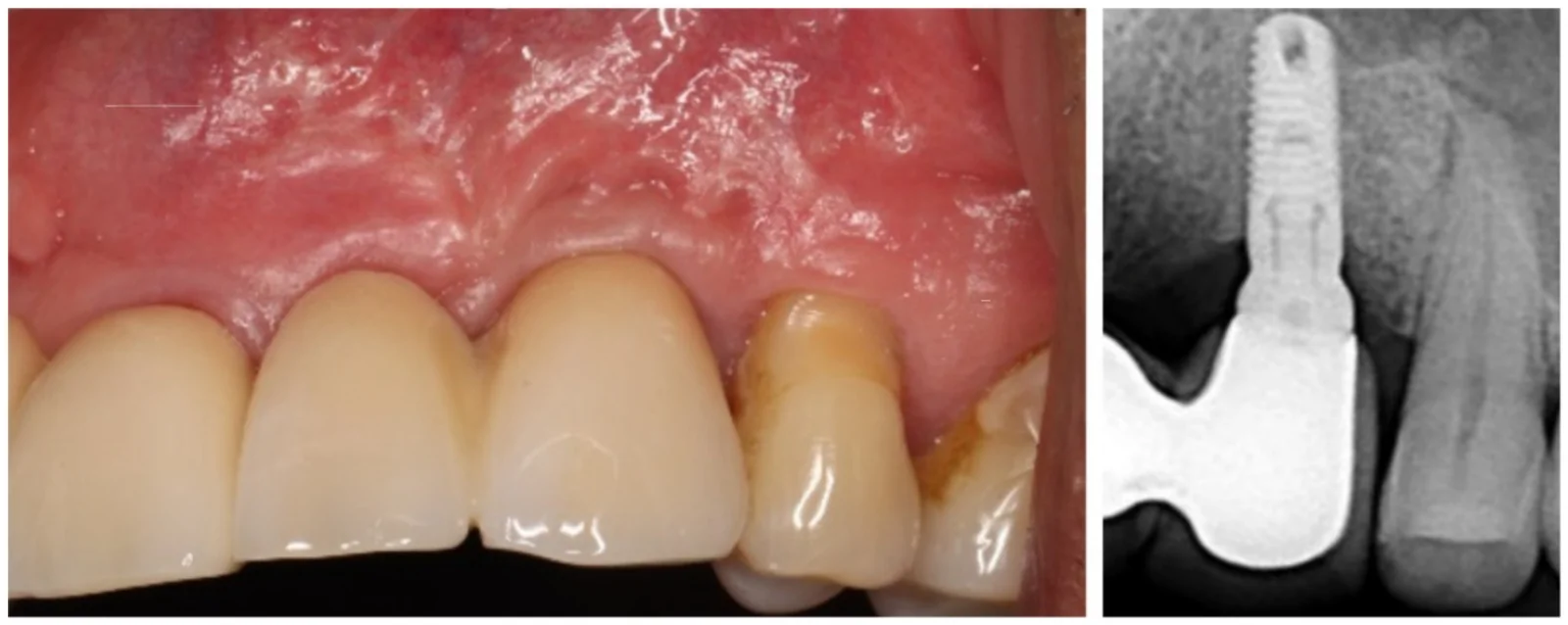
Clinical appearance at three years following restoration (**left**), and corresponding radiographic evaluation (**right**) demonstrating stable peri-implant tissues and maintained bone levels.

A CBCT evaluation conducted four months postoperatively demonstrated substantial regeneration of the alveolar ridge with favorable ridge width and density (Figure 7). Implant placement was subsequently performed after six months of healing. Adequate bone volume was present, and primary implant stability corresponding to 35 Ncm was achieved (Figure 8). Following a six-month osseointegration period, restorative treatment was initiated. Clinical and radiographic follow-ups demonstrated stable peri-implant hard and soft tissues with maintenance of ridge contours and esthetics (Figure 9 and Figure 10).

## 3. Case 2

**Figure 11:**
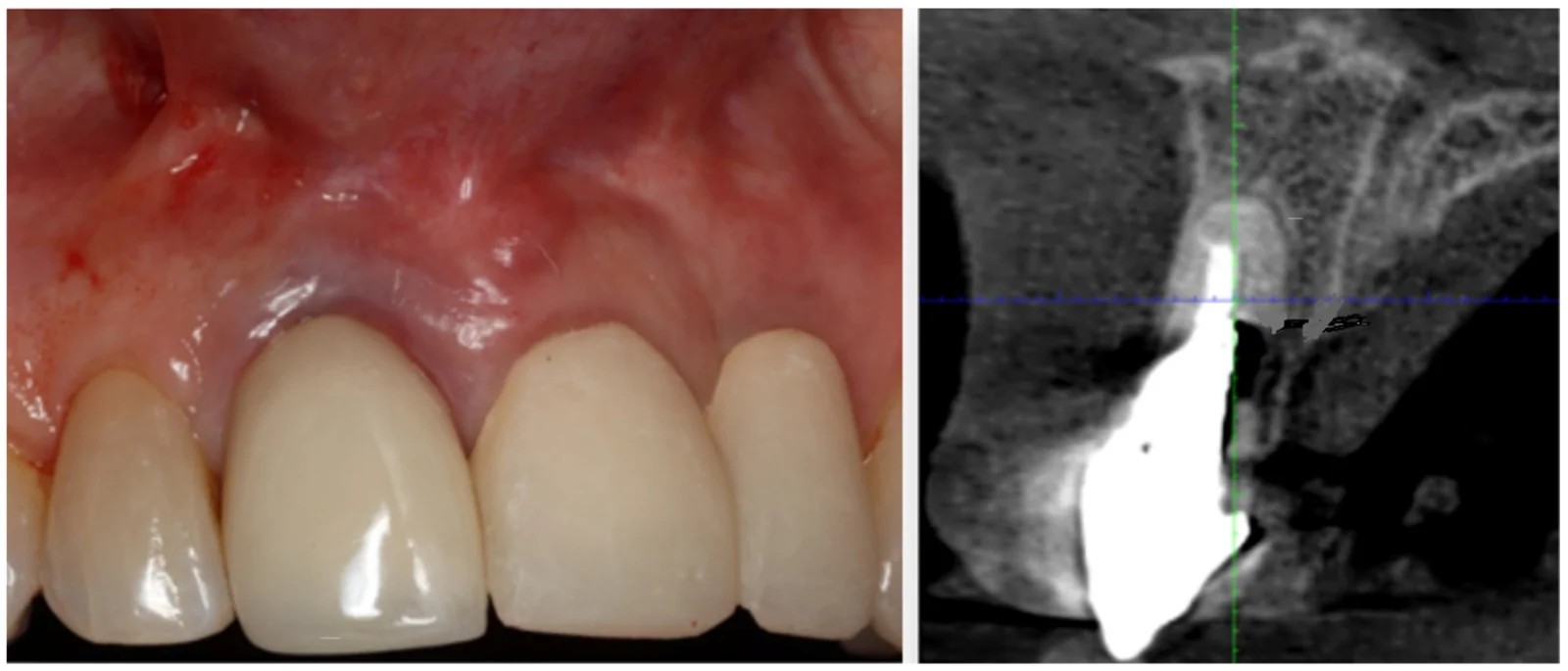
Initial clinical presentation demonstrating discoloration and soft-tissue shadowing associated with the maxillary right central incisor (**left**). CBCT cross-sectional image demonstrating extensive buccal plate loss and root dehiscence (**right**).

A 45-year-old female presented with pain and mobility associated with the maxillary right central incisor. The tooth had previously undergone endodontic treatment and had been restored with a cast post and porcelain-fused-to-metal crown (Figure 11). CBCT evaluation revealed extensive loss of the buccal plate and partial loss of the palatal plate, resulting in a significant osseous defect (Figure 11).

**Figure 12:**
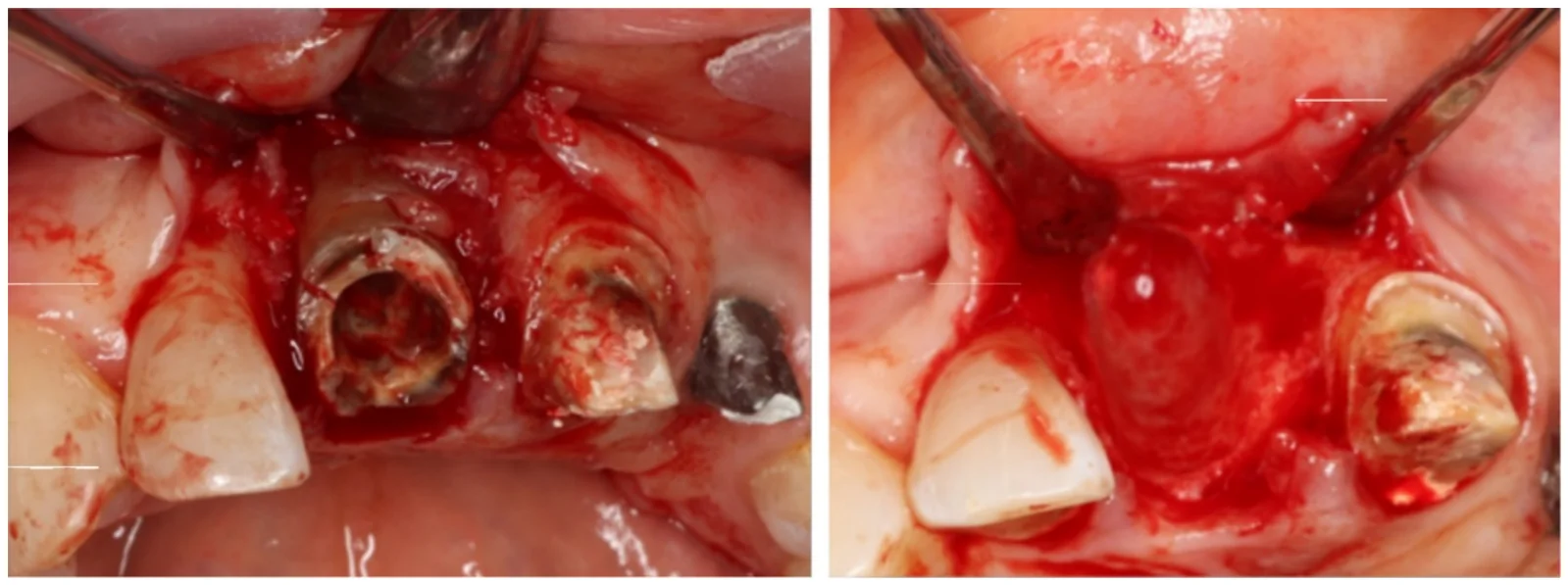
Surgical exposure following removal of existing restorations demonstrating absence of the buccal plate over the root surface (**left**). Extraction socket following tooth removal with complete loss of the facial plate (**right**).

Following flap elevation and atraumatic extraction, complete loss of the buccal plate was confirmed clinically (Figure 12). The extraction socket was thoroughly debrided and irrigated. A connective-tissue graft was harvested from the palate to enhance soft-tissue thickness and support esthetic outcomes. A 3.5 × 13 mm implant was placed in a prosthetically guided position. The residual peri-implant defect was grafted with a combination of a cortical–cancellous allograft and a mineralized/demineralized cortical allograft.

**Figure 13:**
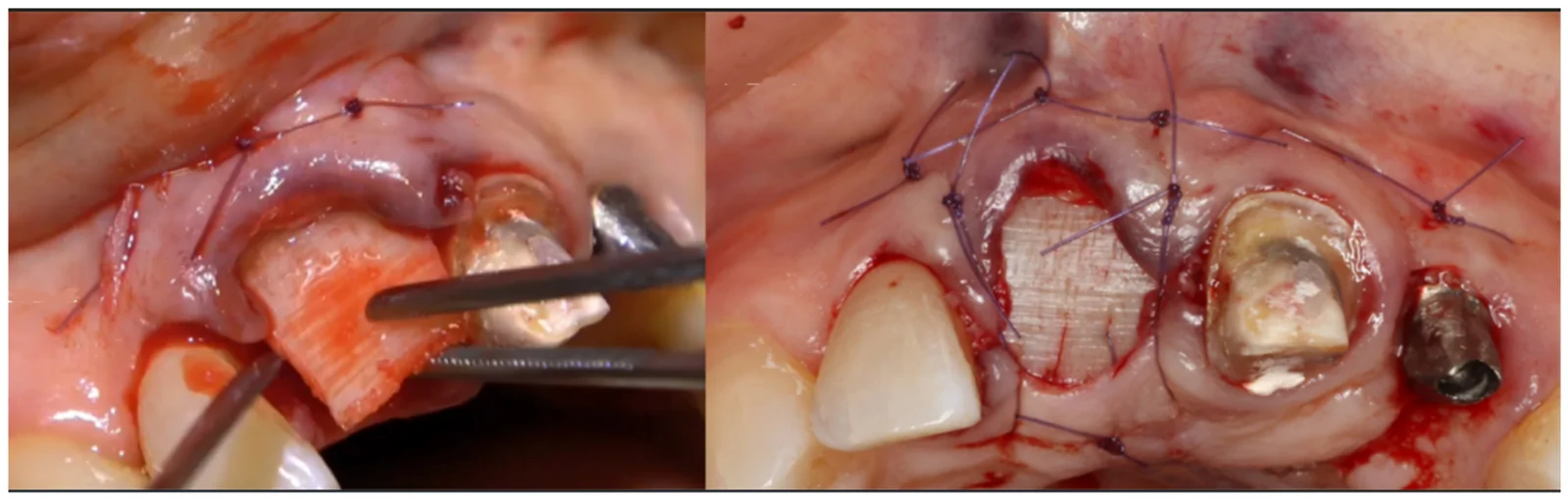
Adaptation of flexible allogeneic laminar bone over the grafted implant site (**left**). Stabilization of the grafted site and flap closure following regenerative treatment (**right**).

Flexible allogeneic laminar bone was adapted beneath the flap and secured to the palatal tissues to contain the particulate graft and maintain regenerative space (Figure 13). A bonded provisional restoration was fabricated and attached to adjacent teeth to seal the socket and support peri-implant soft-tissue architecture throughout healing.

**Figure 14:**
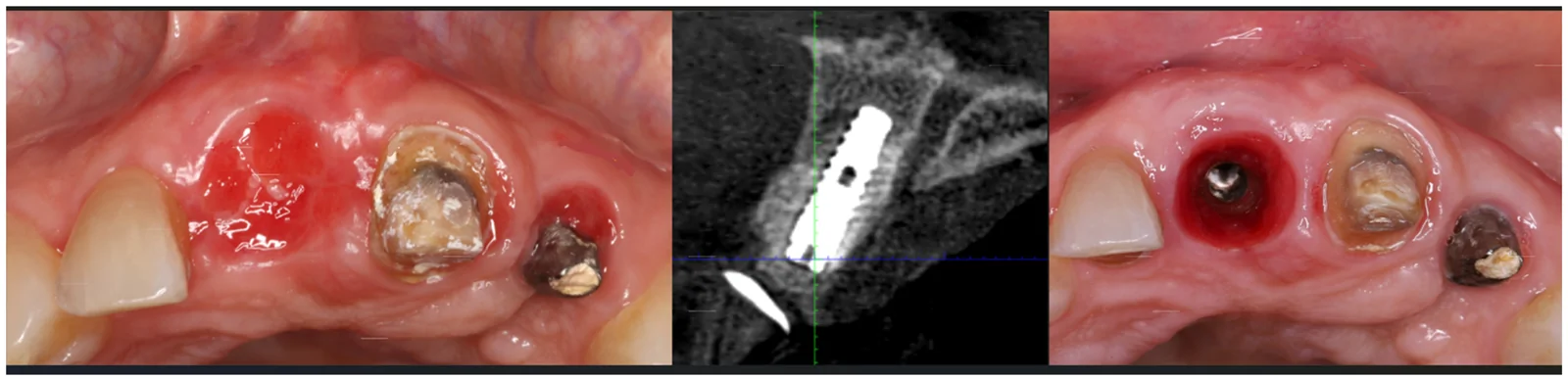
Clinical appearance at 45 days post-operation demonstrating favorable soft-tissue healing (**left**). Four-month CBCT image showing regenerated bone surrounding the implant (**middle**). Implant exposure at six months before restorative treatment (**right**).

**Figure 15:**
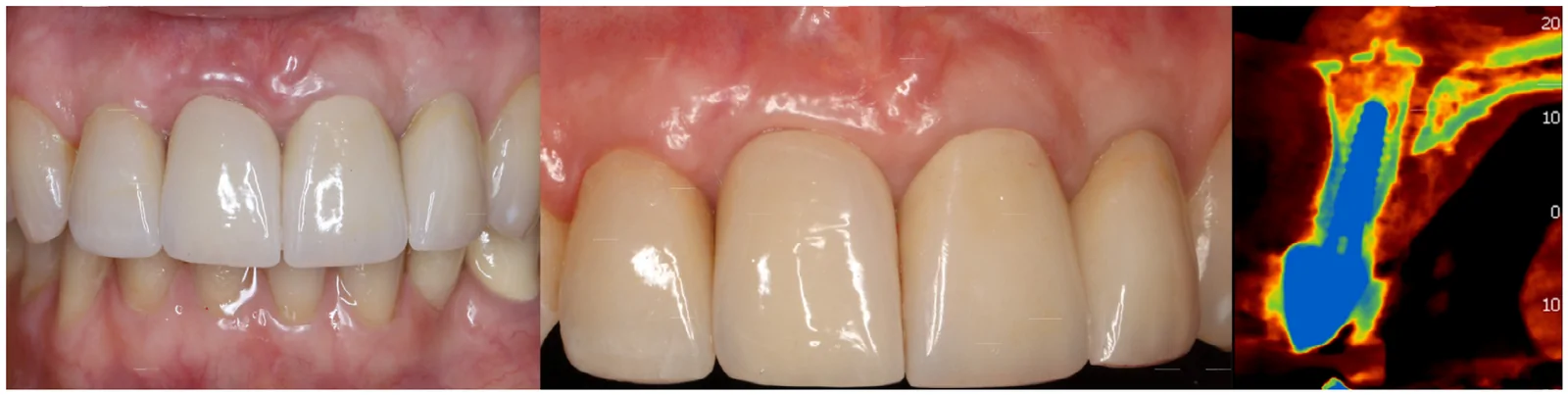
Definitive implant-supported restoration at delivery (**left**). Three-year clinical follow-up demonstrating stable soft-tissue contours (**middle**). CBCT evaluation confirming maintenance of regenerated buccal bone, colors indicate variances in density (**right**).

At 45 days postoperation, soft-tissue healing was uneventful, with excellent epithelialization and preservation of ridge contours (Figure 14). A four-month CBCT evaluation demonstrated bone formation around the implant and restoration of the buccal ridge profile. After six months of healing, the implant was uncovered and restored with a provisional crown to sculpt the peri-implant soft tissues. Following two months of tissue maturation, the definitive restoration was established (Figure 15).

The three-year follow-up demonstrated stable peri-implant soft tissues, preservation of ridge contours, and maintenance of buccal bone volume, as confirmed clinically and radiographically.

## 4. Case 3

**Figure 16:**
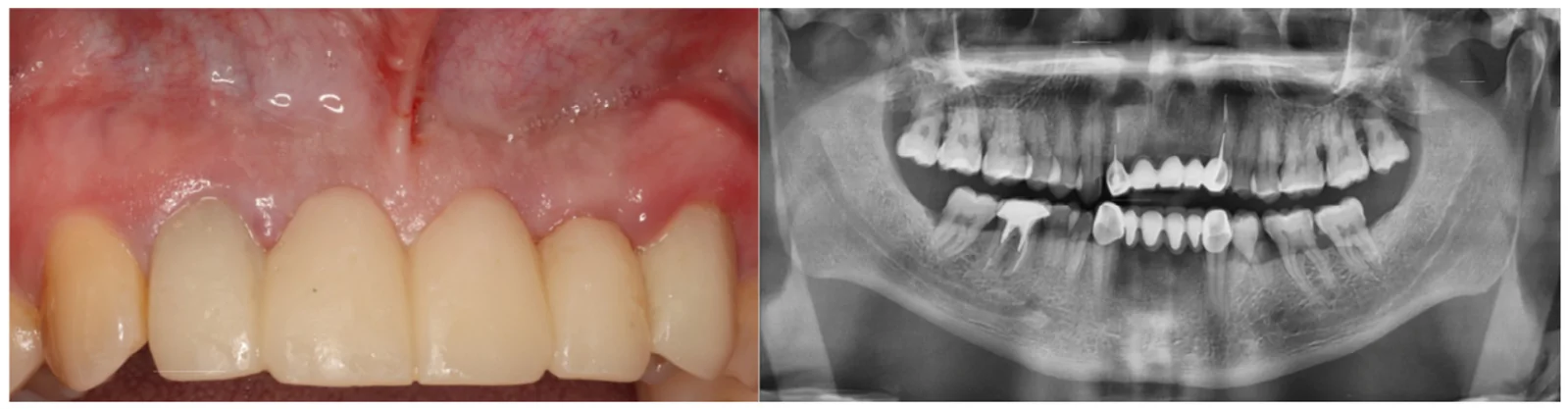
Initial clinical presentation of an anterior fixed partial denture extending from the maxillary right lateral incisor to the maxillary left canine (**left**). Panoramic radiograph demonstrating compromised abutment teeth and ridge deficiency (**right**).

A 45-year-old male presented with a fixed partial denture extending from the maxillary right lateral incisor to the maxillary left canine. Clinical and radiographic examination revealed a moderately atrophic anterior maxillary ridge with significant horizontal deficiency in the edentulous segment (Figure 16).

**Figure 17:**
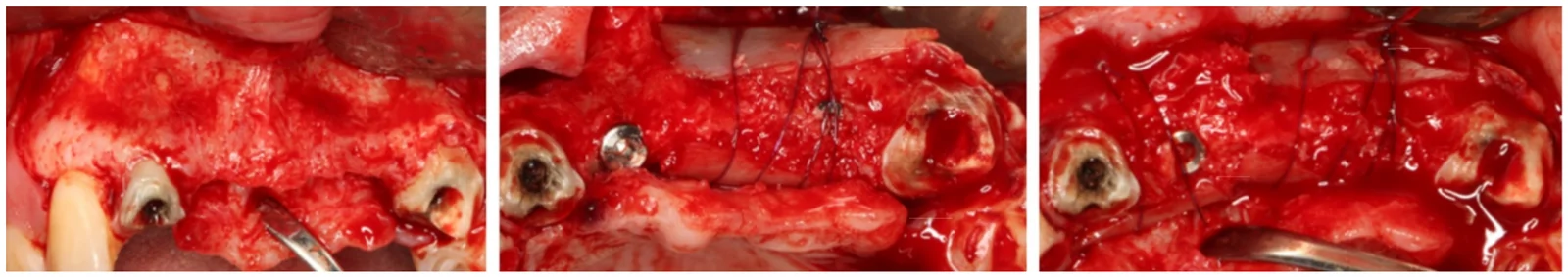
Ridge deficiency following removal of the fixed partial denture (**left**). Placement of particulate graft and flexible laminar bone with stabilization using resorbable sutures ((**middle**) and (**right**)).

Following removal of the existing prosthesis, a full-thickness flap was elevated to expose the deficient ridge (Figure 17). Two implants were placed in the maxillary right central incisor and left lateral incisor positions. Ridge augmentation was performed using a mixture of a mineralized/demineralized cortical allograft and a cortical–cancellous allograft. Flexible allogeneic laminar bone was adapted over both the buccal and palatal aspects of the grafted region to ensure containment and maintain the desired ridge contours.

**Figure 18:**
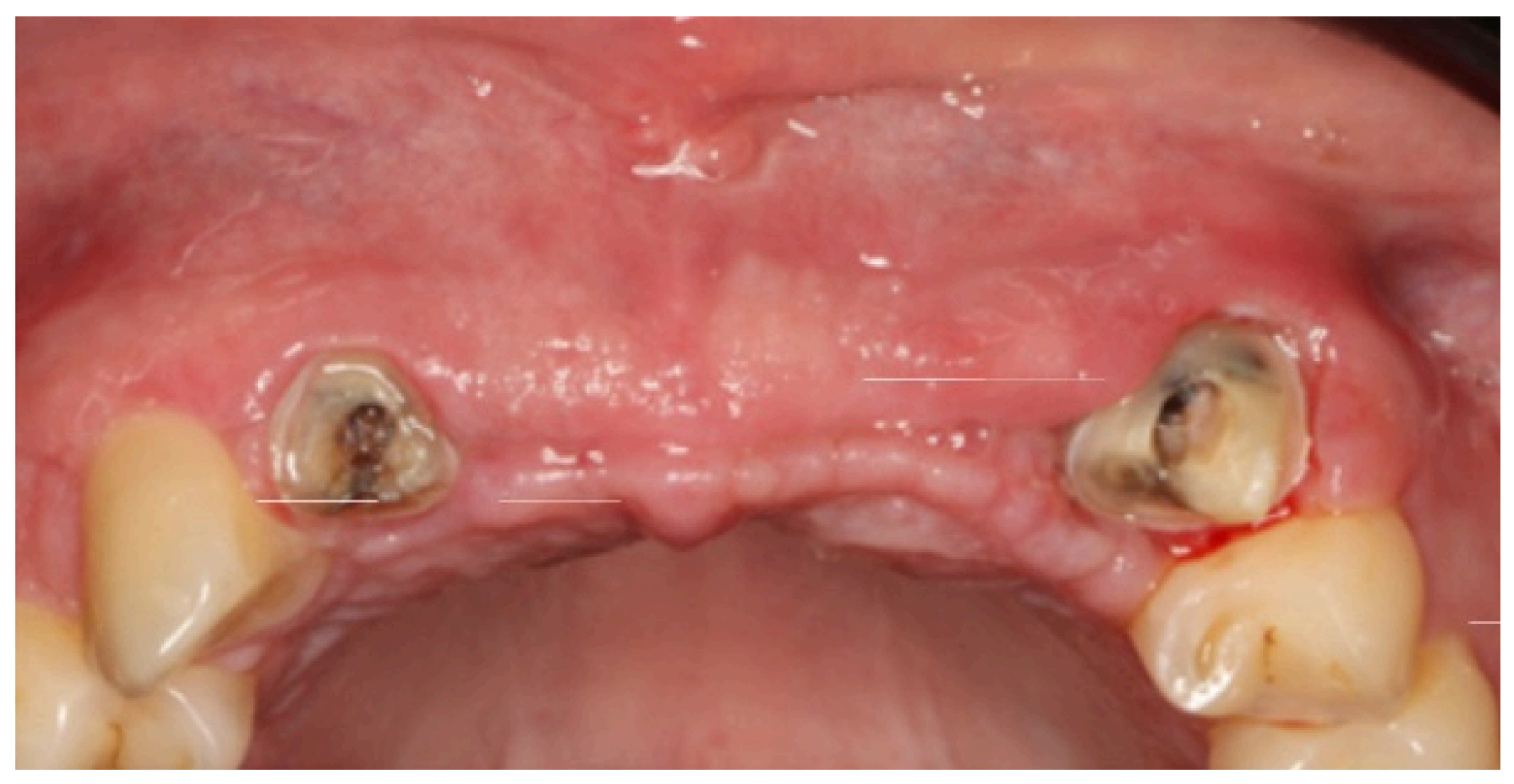
Four-week postoperative healing demonstrating preservation of the ridge contour and favorable soft-tissue adaptation.

The laminar bone was stabilized with periosteal sutures, and primary flap closure was achieved without tension (Figure 17). Soft-tissue healing was uneventful, and favorable ridge contours were evident at four weeks post-operation (Figure 18).

**Figure 19:**
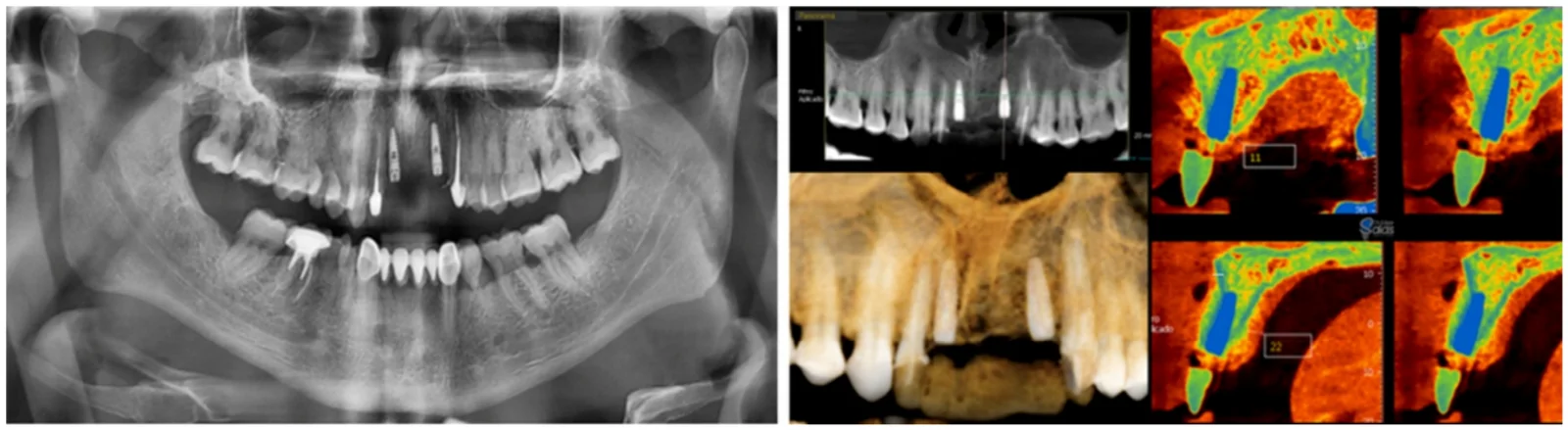
Four-month postoperative panoramic radiograph (**left**) and CBCT images ((**middle**) and (**right**)) demonstrating successful ridge augmentation and bone formation around the implants, colors indicate variances in density.

**Figure 20:**
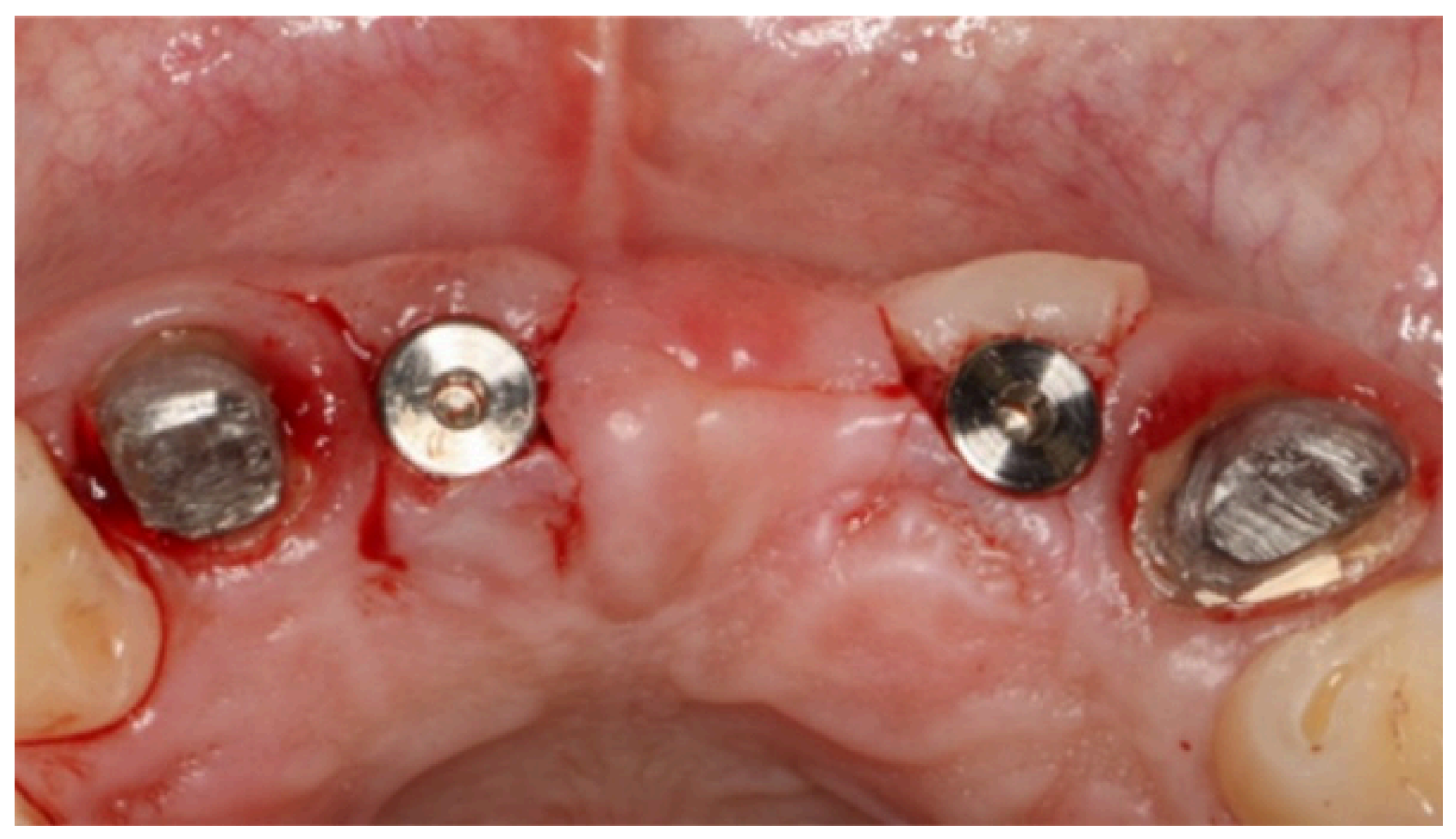
Second-stage implant surgery performed following six months of healing.

Four-month follow-up radiographs and CBCT imaging demonstrated successful bone regeneration and maintenance of ridge dimensions around the implants (Figure 19). Second-stage surgery was performed after six months of healing, and provisional restorations were utilized to develop the peri-implant soft tissues (Figure 20).

**Figure 21:**
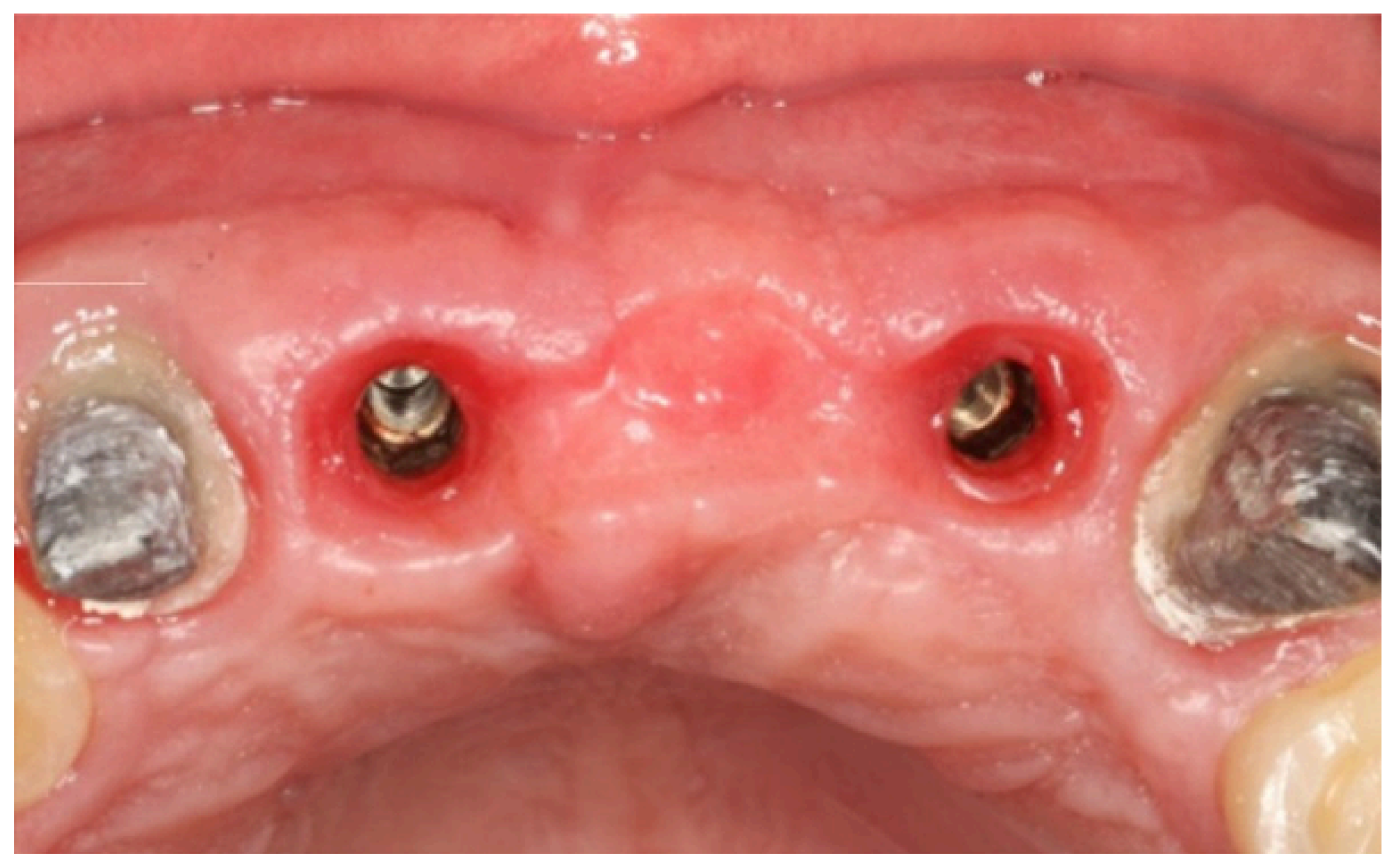
Clinical appearance eight months following augmentation and provisionalization demonstrating a stable ridge contour and favorable peri-implant soft-tissue architecture.

Definitive prosthetic rehabilitation was completed using a three-unit implant-supported restoration. Clinical evaluation demonstrated harmonious soft-tissue contours, adequate ridge volume, and satisfactory esthetic integration with the adjacent dentition (Figure 21).

## 5. Discussion

Successful implant therapy depends on whether there is enough hard and soft tissue to permit prosthetically driven implant placement and long-term maintenance of peri-implant health. Following tooth extraction, physiological remodeling of the alveolar process frequently results in significant horizontal and vertical bone loss, particularly along the buccal aspect of the ridge. These dimensional changes can compromise implant positioning, esthetic outcomes, and long-term restorative success. Consequently, ridge preservation and augmentation procedures have become integral components of contemporary implant therapy.

Guided bone regeneration remains one of the most predictable approaches to treating localized ridge deficiencies. The biological principle of GBR is based on the exclusion of rapidly proliferating soft-tissue cells from the regenerative site while maintaining a protected space for the migration of osteogenic cells and vascularization. Although traditional non-resorbable membranes and titanium mesh have demonstrated predictable outcomes, their use is associated with several disadvantages, including increased surgical complexity, membrane exposure, patient discomfort, and a frequent requirement for a second surgical procedure for removal [13,14].

Flexible allogeneic laminar bone is a biological alternative that combines the functions of a containment device and a slowly remodeling scaffold. Unlike conventional collagen membranes, which primarily function as passive barriers, flexible allogeneic laminar bone provides semi-rigid structural support that assists in maintaining regenerative space during healing. This characteristic may be particularly advantageous in regard to horizontal ridge defects where preservation of ridge contours is essential for optimal implant placement and esthetic outcomes [15,16,17,18].

The material’s flexibility permits intimate adaptation to irregular osseous contours without fracturing while maintaining sufficient rigidity to resist collapse of the overlying soft tissues. In this case series, flexible allogeneic laminar bone effectively contained the particulate graft material and maintained ridge dimensions throughout the healing period. Clinical and radiographic evaluations demonstrated successful augmentation of the deficient ridges and the development of sufficient bone volume to permit implant placement in prosthetically ideal positions. However, because standardized volumetric CBCT measurements, objective ridge-width analyses, and quantitative assessments of horizontal bone gain were not performed, these observations should be interpreted as qualitative clinical and radiographic findings rather than objective measurements of regenerative efficacy.

An additional advantage of flexible allogeneic laminar bone is that it eliminates donor-site morbidity associated with autogenous block grafting procedures. Although autogenous bone remains the reference standard because of its osteogenic potential, harvesting procedures increase surgical time, postoperative discomfort, and the risk of donor-site complications [19]. The use of allogeneic materials allows clinicians to avoid these disadvantages while still achieving clinically acceptable regenerative outcomes [20,21,22,23]. Furthermore, the material can easily be trimmed and adapted to complex ridge morphologies, reducing surgical complexity relative to autogenous block grafting while providing greater structural support than conventional collagen membranes [15,16,17,18,19,20,21,22,23].

The combination of flexible allogeneic laminar bone with particulate mineralized and demineralized allografts may exert a synergistic regenerative effect. The particulate graft functions as an osteoconductive scaffold that supports cellular migration and vascular ingrowth, while the laminar bone serves as a biological containment structure that stabilizes the graft and preserves regenerative space. Together, these materials create a favorable environment for new-bone formation and ridge reconstruction [15,20,21,22,23].

The favorable outcomes observed in this series are consistent with previous reports describing the use of allogeneic bone plates and cortical lamina techniques for ridge preservation and augmentation. Investigators have reported maintenance of ridge dimensions, favorable handling characteristics, and successful implant placement following healing [15,16,17,18,22]. Furthermore, gradual remodeling of the laminar bone may contribute to physiological integration of the grafted site while reducing the long-term complications associated with non-resorbable materials [13,23,24]. Long-term studies have demonstrated stable ridge dimensions and high implant survival more than three to five years after loading, supporting the biologic rationale for using this regenerative approach [15,16,17,18,22,23,24].

A noteworthy clinical observation in the present cases was the maintenance of soft-tissue architecture, particularly in the anterior maxilla, where esthetic demands are greatest. Preservation of vestibular depth and support of the facial soft tissues facilitated development of favorable emergence profiles and harmonious integration of the definitive restorations. These outcomes are particularly important in implant therapy, where soft-tissue stability contributes significantly to long-term esthetic success.

The clinical advantages of flexible allogeneic laminar bone include ease of adaptation to complex ridge morphologies, elimination of donor-site morbidity, reduced surgical complexity relative to autogenous block grafting, and improved space maintenance relative to conventional collagen membranes. In addition, the material functions as both a biological barrier and a slowly remodeling scaffold, potentially reducing the need for secondary procedures associated with non-resorbable membranes and titanium mesh.

As with any regenerative procedure, potential disadvantages and complications should also be considered. Similar to other GBR techniques, premature soft-tissue dehiscence or graft exposure may increase the risk of bacterial contamination, delayed healing, partial graft loss, infection, and reduced regenerative volume. Published studies evaluating cortical allogeneic lamina techniques report overall complication rates ranging from approximately 5% to 20%, with graft exposure rates generally being between 5% and 15%, depending on defect morphology, flap management, and surgical technique [15,16,17,18,22,23,24]. Importantly, many cases of limited exposure of cortical allogeneic laminae can be managed conservatively through meticulous plaque control and local wound care without requiring graft removal. This situation differs from that regarding titanium mesh, for which exposure frequently necessitates premature removal and may compromise the regenerative outcome. Careful flap design, preservation of vascular supply, and tension-free primary closure therefore remain critical to minimizing complications and maximizing regenerative success.

Several limitations should be acknowledged. This investigation represents a descriptive case series involving a limited number of patients and lacks a control group for comparison with alternative augmentation techniques. Although clinical examination, periapical radiographs, and CBCT imaging demonstrated successful ridge reconstruction and implant site development, standardized quantitative measurements of ridge width, horizontal bone gain, volumetric changes, and bone density were not prospectively obtained. Histological evaluation of regenerated tissues was also unfeasible. Consequently, definitive conclusions regarding superiority over other regenerative approaches cannot be drawn, and the findings should be considered preliminary clinical observations. Future prospective controlled studies incorporating calibrated CBCT-based linear and volumetric analyses, implant stability measurements, patient-reported outcomes, and, when feasible, histologic evaluation must be conducted to more objectively determine the effectiveness, predictability, and long-term stability of flexible allogeneic laminar bone in ridge augmentation procedures.

## 6. Conclusions

Within the limitations of this three-patient case series, flexible allogeneic laminar bone represents a promising adjunct for guided bone regeneration procedures involving implant site development. In the cases presented, the material ensured effective containment of particulate allografts, maintenance of regenerative space, and preservation of ridge contours sufficient to permit prosthetically driven implant placement.

The combination of flexible laminar bone and particulate allografts was associated with favorable clinical and radiographic outcomes while avoiding the donor-site morbidity associated with autogenous block grafting and the additional surgical procedures often required with non-resorbable barrier systems. Long-term follow-ups demonstrated stable peri-implant hard- and soft-tissue conditions and maintenance of the reconstructed ridge architecture in these patients.

Although these findings are encouraging, they should be interpreted as preliminary clinical observations rather than definitive evidence of treatment efficacy or superiority over established augmentation techniques. Larger prospective controlled clinical studies incorporating standardized quantitative CBCT measurements, volumetric analyses, objective assessment of ridge width changes, patient-reported outcomes, and long-term follow-ups are required to further evaluate the predictability, comparative effectiveness, and long-term stability of this regenerative approach.

## Data Availability

The raw data supporting the conclusions of this article will be made available by the authors on request.
